# Pan‐Epigenetic Age Prediction in Mammals

**DOI:** 10.1111/acel.70380

**Published:** 2026-01-27

**Authors:** Zane Koch, Adam Li, Trey Ideker

**Affiliations:** ^1^ Program in Bioinformatics and Systems Biology University of California San Diego La Jolla California USA; ^2^ Department of Bioengineering University of California San Diego La Jolla California USA; ^3^ Department of Medicine University of California San Diego La Jolla California USA

## Abstract

Epigenetic remodeling is a hallmark of aging, yet which epigenetic layers are most affected during aging—and the extent to which they are interrelated—is not well understood. Here, we perform a comprehensive analysis of epigenetic aging encompassing 6 histone marks and DNA methylation measured across 12 tissues from > 1000 humans and mice. We identify a synchronized pattern of age‐related changes across these epigenetic layers, with all changes converging upon a common set of genes. Notably, an epigenetic clock based on these genes can accurately predict age using data from any layer (Spearman *ρ*: 0.70 in humans, 0.81 in mice). Applying this “pan‐epigenetic” clock, we observe that histone modification and DNA methylation profiles agree in the prediction of which individuals are aging more rapidly or slowly. These results demonstrate that epigenetic modifications are subject to coordinated remodeling over the lifespan, offering a unified view of epigenetic aging.

## Graphical abstract

Across > 1000 human and mouse epigenomes, histone modifications and DNA methylation undergo coordinated, cross‐tissue remodeling at shared genes.

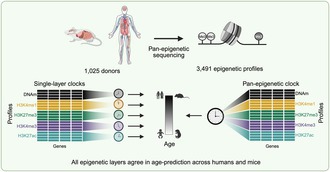

## 1 Introduction

Among the hallmarks of aging, considerable attention has been devoted to the study of epigenetic alterations, particularly changes in DNA methylation patterns (Gonzalo 2010). Across the human lifespan, thousands of cytosine‐guanine dinucleotides (CpGs) undergo systematic gains or losses of methylation (Christensen et al. 2009; Fraga et al. 2005; Hannum et al. 2013; Li et al. 2022). These widespread shifts have enabled the development of robust methylation‐based age predictors, termed “epigenetic clocks” (Belsky et al. 2022; Hannum et al. 2013; Horvath 2013; Levine et al. 2018; Lu et al. 2019), which have been used pervasively in clinical and forensic applications (Bell et al. 2019; Higgins‐Chen et al. 2022; Paparazzo et al. 2023). Remarkably, these age‐related methylation changes are highly conserved across diverse tissues and species (Wang et al. 2020), such that a single epigenetic clock can predict the age of over 100 distinct mammalian species (Lu et al. 2023).

DNA methylation (DNAm) represents only one facet of the epigenome; however, another major class of epigenetic mark relates to chemical modifications of histones (Geiman and Robertson 2002), which regulate chromatin structure, DNA accessibility, and gene expression. The number of distinct types of histone marks is vast and includes histone methylation, acetylation, and phosphorylation at multiple sites—some of which have known functions while many others remain incompletely understood (Zhao and Garcia 2015). For instance, monomethylation of lysine 4 of histone 3 (H3K4me1) commonly marks enhancers poised for activation (Spicuglia and Vanhille 2012), whereas H3K27me3 is associated with heterochromatic domains and transcriptional repression (Cai et al. 2021). The occupancy of particular histone marks across the genome has been extensively investigated via chromatin immunoprecipitation sequencing (ChIP‐seq), which uses DNA sequencing to read out the genomic regions that co‐precipitate with a particular modified histone (Park 2009). The combined influence of histone modifications and DNA methylation ultimately determines chromatin accessibility—the physical openness of chromatin to transcription factors and regulatory proteins (Klemm et al. 2019).

Recent studies suggest that age‐related changes occur not only in CpG methylation (Chien et al. 2024; Johnson et al. 2020; Occean et al. 2024), but in some of these other epigenetic layers including histone modifications (de Lima Camillo et al. 2025; Greer et al. 2010; Maures et al. 2011; Sen et al. 2015) and chromatin accessibility (Bozukova et al. 2022; Rechsteiner et al. 2022). Such multifaceted change aligns with the concept that the various epigenetic processes form a tightly interconnected regulatory network, whereby alterations in one layer could reverberate through others (Fu et al. 2020). Outside of the aging field, general studies of histone marks and DNA methylation have shown that these layers exert reciprocal effects on one another (Fu et al. 2020), driven by direct physical interactions and the shared regulatory activity of DNA methyltransferases and histone‐modifying enzymes (Lehnertz et al. 2003; Zhang et al. 2010). In studies of aging, however, each type of epigenetic mark has typically been analyzed in isolation without relation to other epigenetic layers.

Here, we explore to what extent the lifetime changes seen in DNA methylation and histone modifications are interrelated. By analyzing epigenetic profiles from hundreds of humans and mice, we observe that the different epigenetic layers show coordinated changes during aging, with changes in all layers converging on a common set of genes. We find that an epigenetic clock based on these genes can predict age using data from any epigenetic layer, with predictions from each layer agreeing in which individuals are aging more slowly or more rapidly.

## 2 Results

### 2.1 Epigenetic Layers Show Coordinated Changes During Aging

**FIGURE 1:**
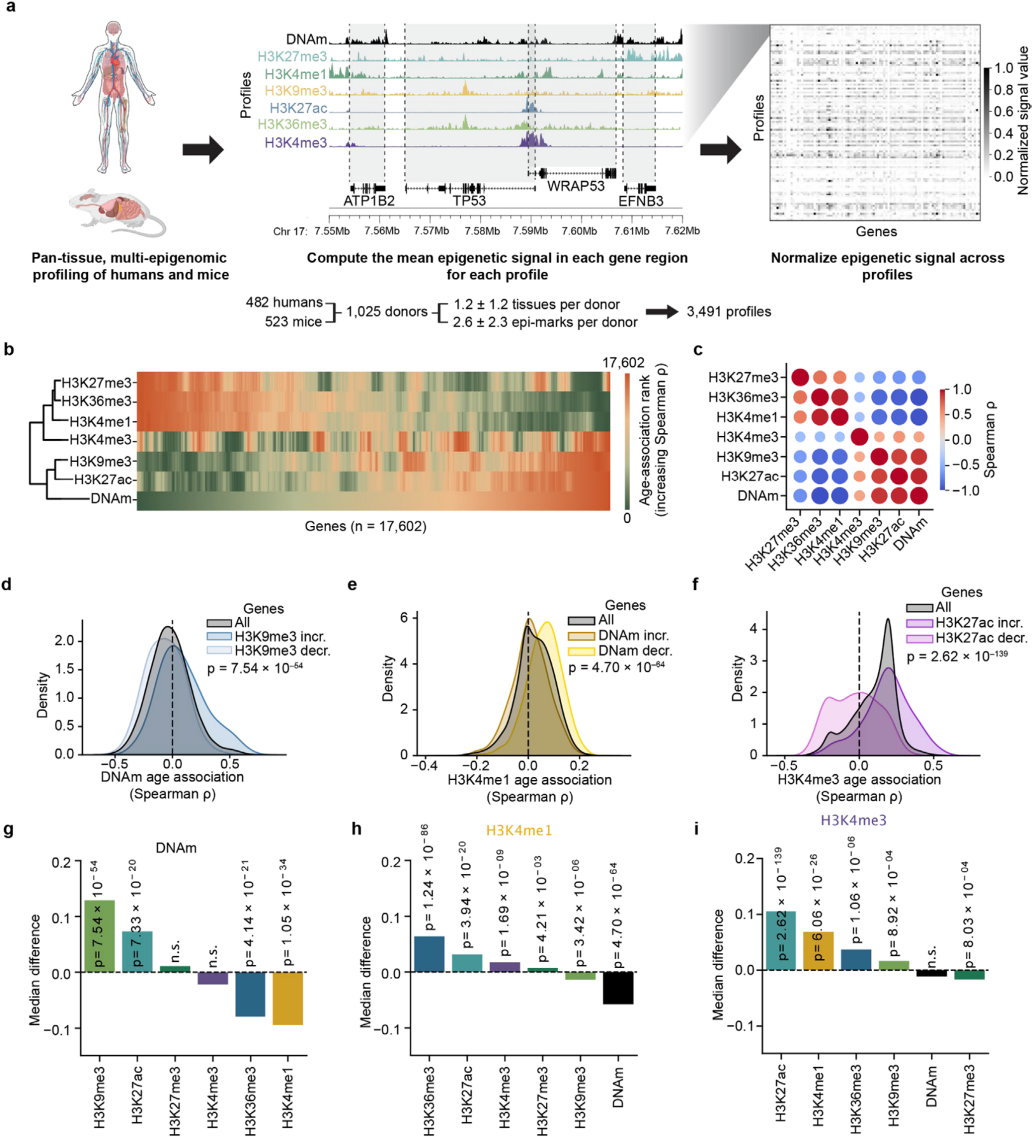
Common epigenetic remodeling during aging. (a) Workflow for generating gene‐level epigenetic features. The mean signal value within each gene region for profiles from human and mouse donors was calculated, batch corrected, and normalized across profiles to create a feature matrix. (b) Heatmap of the association (Spearman's *ρ*) between the signal of each epigenetic layer and age across 17,602 genes (*n* = 482 human donors). Genes (columns) are ordered by increasing DNA methylation age‐association and colored by the rank of association within each layer, with green denoting the most negative correlations and orange the most positive. (c) Heatmap of pairwise Spearman correlations (*ρ*) between gene‐level age associations for each epigenetic mark; all correlations were statistically significant (*p* < 1 × 10^−10^, two‐sided Spearman correlation test). Circle size and color represent the strength and direction of correlations. (d) Kernel density estimate (KDE) of Spearman's *ρ* between DNA methylation and age for all genes (gray) versus the 1000 genes with the most positive (light color) or most negative (dark color) H3K9me3 age associations. *p*‐value was calculated based on a two‐sided Mann–Whitney *U* (MWU) test comparing the distribution of DNAm age‐association values between the sets of positively and negatively H3K9me3 age‐associated genes. (e) Similar to (d), but stratifying H3K4me1 age‐associations based on DNAm levels. (f) Similar to (d), but stratifying H3K4me3 age‐associations based on H3K27ac levels. (g‐i), For each focal mark—DNAm (g), H3K4me1 (h), and H3K4me3 (i)—genes were ranked by their Spearman age‐association (*ρ*) according to each of the other epigenetic layers (*x*‐axis) and split into the 1000 most positively and 1000 most negatively associated. Bars show the median difference in *ρ* of the focal mark between these two gene sets. *p* values (two‐sided Mann–Whitney *U* test) are shown above each bar; “n.s.” denotes *p* ≥ 0.05.

To characterize epigenetic change during aging, we collected histone modification (ChIP‐seq) and DNA methylation (whole‐genome bisulfite sequencing; WGBS) profiles from nine previous studies (Bujold et al. 2016; ENCODE Project Consortium et al. 2020; Fernández et al. 2016; Hillje et al. 2022; Meer et al. 2018; Petkovich et al. 2017; Signal et al. 2024; Stubbs et al. 2017; Yang, Occean, et al. 2023). These 3491 profiles were drawn from 482 humans and 523 mice with each individual donor contributing profiles from one or more of seven epigenetic layers across up to twelve tissues (average 3.5 profiles per donor; Figure S1). Each histone profile reflected the proportion of cells bearing that particular histone modification at each genomic locus. For DNAm, each profile encoded the percent of cells in the measured tissue for which each CpG was methylated. To harmonize features across studies, all measured sites within the same gene body were pooled and their signal averaged to produce a single continuous value per epigenetic layer per gene (Figure 1a; Methods).

Correlating the epigenetic signal in each gene with age, we found that certain epigenetic layers exhibited more age‐related change than others. For H3K27me3, we observed a significant association of the occupancy of this epigenetic mark with age for 77.3% of human genes, whereas for H3K9me3, a significant age association was seen for only 0.6% of genes (Figure S2a,b). Nevertheless, there was a clear coordination between the age‐related changes among epigenetic layers, with repressive marks H3K27me3, H3K9me3, and DNA methylation aligned in their direction of change, and activating marks (H3K36me3, H3K4me1) exhibiting an opposing pattern (Figure 1b,c). Unexpectedly, while H3K27ac and H3K4me3 had been largely associated with the activation of transcription, in our aging study they showed a modest but significant trend in the same direction as the repressive marks (Figure 1c). Overall, we found that for 12 of the 21 possible pairs of layers, the direction of age‐related change between the two layers was associated (Figures 1d–i and S3a–d). For instance, DNA hypomethylation co‐occurred with a loss of H3K9me3, whereas the accrual of H3K9me3 co‐occurred with DNA hypermethylation (Figure 1d). In contrast, H3K4me1 and DNAm exhibited the opposite relationship, with an increased deposition of one associating with a loss of the other (Figure 1e).

### 2.2 Changes in All Epigenetic Layers Converge Upon a Common Set of Genes

**FIGURE 2:**
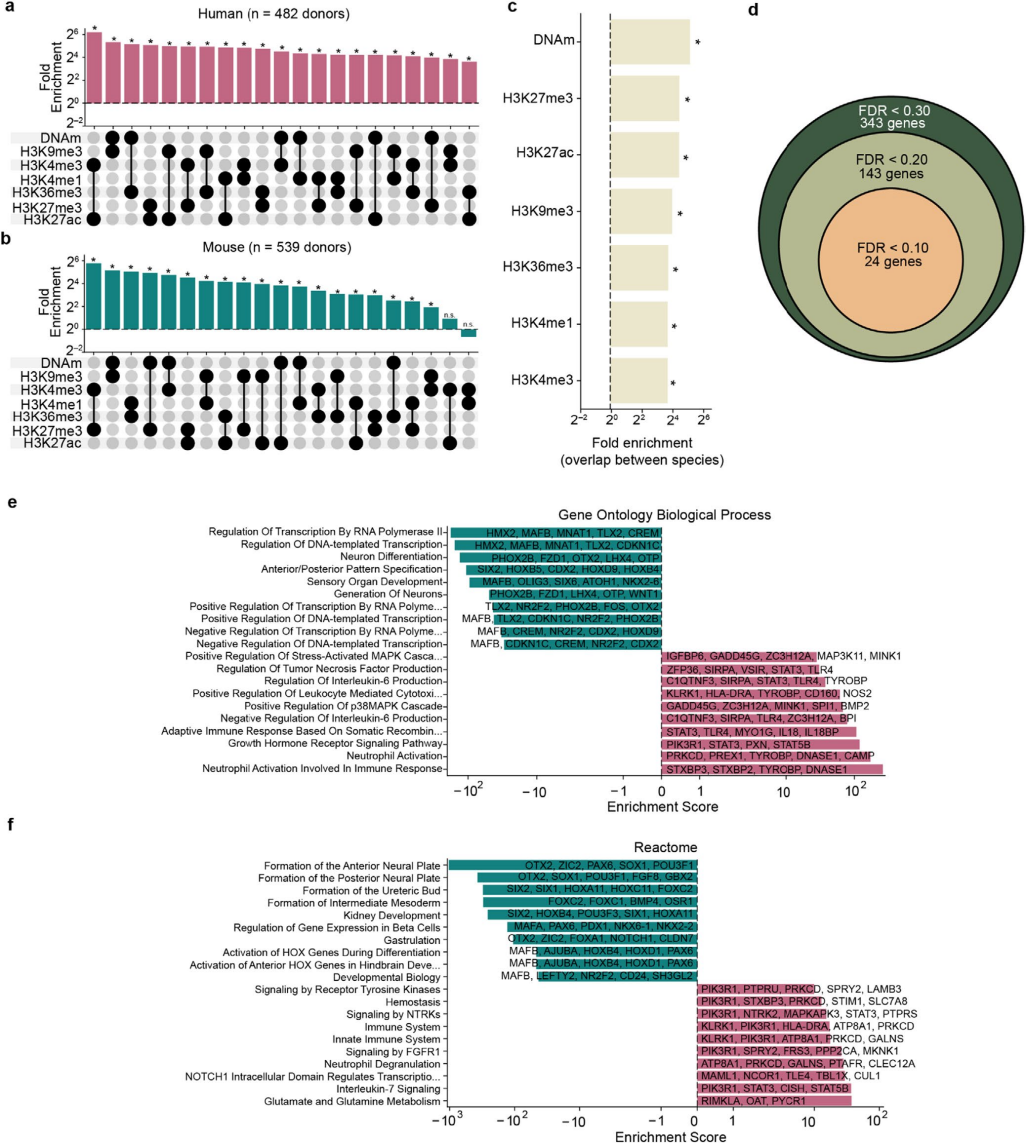
Overlap among genes exhibiting age‐related epigenetic change. (a) Upset plot showing the fold enrichment for the overlap between the top 1000 most age‐associated human genes (absolute Spearman's *ρ*) for each pair of epigenetic marks compared to random expectation (*n* = 482 human donors; Methods). (*) indicates *p* < 0.005 based on a two‐sided binomial test. (b) Similar to (a), but for age‐associations calculated in mice (*n* = 523 mouse donors). (c) Bar plot depicting the fold enrichment of the top 1000 age‐associated genes for each epigenetic mark between mice and humans. (*) indicates *p* < 5.0 × 10^−36^ based on a two‐sided binomial test. (d) Nested circles representing the number of genes found to be significantly associated with age in all seven epigenetic layers at three different FDR thresholds (*n* = 482 human donors). The radius of each circle is proportional to log_10_ of the number of genes represented by that circle. (e) Gene Ontology Biological Process enrichment scores for genes showing the strongest epigenetic repression (teal bars, left of zero) versus activation (magenta bars, right of zero) with age. Processes are ordered by log‐scaled enrichment score on the *x*‐axis, with increased enrichment indicated by values far from zero. The 10 up‐ and down‐regulated pathways with the largest enrichment score are shown. All enrichments are significant (*p* < 0.05). Representative genes contributing to each term are listed alongside each bar. (f) Reactome pathway enrichment plotted as in (e).

We found that the genes exhibiting the strongest associations with age were largely consistent across epigenetic layers (Figure 2a). For example, the genes for which DNA methylation state was most correlated with age were highly likely to be the same genes for which H3K9me3 occupancy was correlated with age (34‐fold enrichment, *p* = 5.5 × 10^−120^; Figure 2a). This same convergence among epigenetic layers was seen in mice (Figure 2b) involving largely the same genes as in humans (Figure 2c). In humans, the strongest coordination was seen for H3K4me3 and H3K27ac, while in mice H3K4me3 and H3K27me3 were most tightly coupled, although nearly all epigenetic layers showed significant convergence on a common set of genes in both species. At a false discovery rate (FDR) of 20%, a total of 143 genes exhibited age‐related changes across all seven epigenetic layers (Figure 2d).

Genes exhibiting the greatest epigenetic repression with age—those that accumulated repressive marks and lost activating marks—were highly enriched for developmental functions related to HOX gene expression and organ formation (Figure 2e,f; Methods). Such genes included *SOX1*, *WNT1*, and *HOXB5*. In contrast, genes with the greatest epigenetic activation with age were related to inflammatory pathways (Figure 2f), including *SIPRA* and *CAMP*.

Taken together, these analyses (Figures 1 and 2) indicated that aging is accompanied by a coordinated shift in histone modifications and DNA methylation, with both activating and repressive marks changing together at shared genomic loci. While the direction of change varied by epigenetic mark, the loci that underwent the largest modifications during aging were conserved across species, suggestive of an intertwined “pan‐epigenetic” process that unfolds over the lifespan.

### 2.3 Comparison of Epigenetic Layers in Quantitative “Clock” Prediction of Age

**FIGURE 3:**
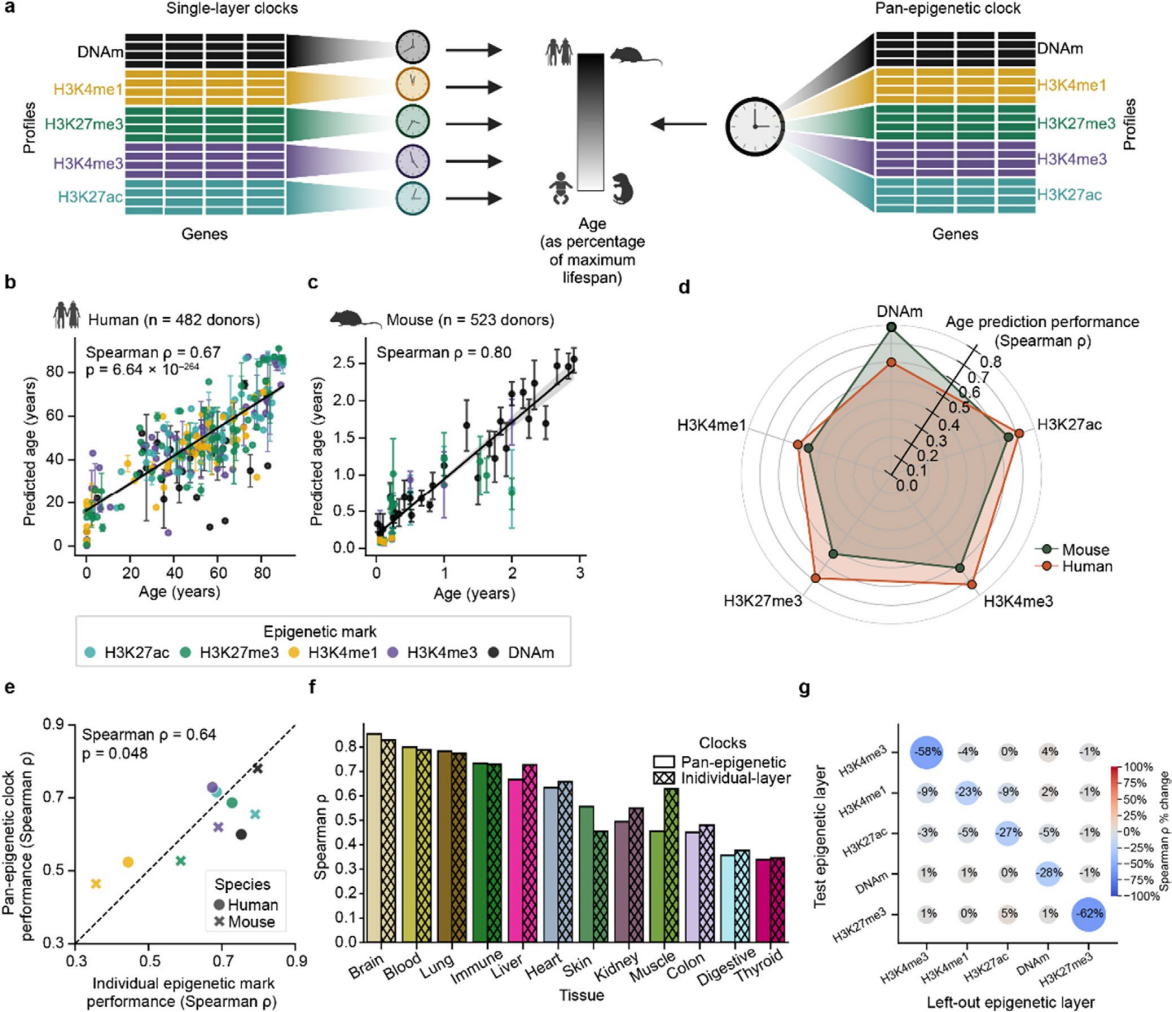
Pan‐epigenetic age prediction in mammals. (a) Two complementary age‐prediction strategies pursued in this study: Five “single‐layer” clocks trained to predict age with a single epigenetic modification (left) versus a unified “pan‐epigenetic” clock trained to predict from any of the five marks (right). Donor ages are represented as a percentage of the species' maximum lifespan. (b) Scatter plot of predicted versus actual age for human donors (*n* = 482 donors, *n* = 2029 profiles) using the pan‐epigenetic clock, evaluated on held‐out donors in 10‐fold cross‐validation. Each point represents an individual donor and error bars represent the standard deviation of age predictions across samples from each tissue profiled in that donor. Predictions based on different epigenetic marks are shown in different colors. (c) Similar to (b) but for mouse donors (*n* = 523 mice, *n* = 569 profiles). (d) Radar plot comparing the predictive performance (Spearman *ρ*) of the pan‐epigenetic clock on samples of each epigenetic modification and species (human: Orange polygon, *n* = 482 donors; mouse: Gray polygon, *n* = 523 donors). (e) Scatter plot comparing the predictive performance of the single‐layer clocks to that of the pan‐epigenetic clock. Each point represents the Spearman correlation between predicted and actual age for a particular epigenetic mark (indicated by color, same as panel b) in a particular species (indicated by shape). (f) Spearman correlation between predicted and actual age in each tissue (human: *N* = 482 donors; mouse: *N* = 523 donors). Bars denote the Spearman *ρ* across all epigenetic mark types based upon the pan‐epigenetic clock (solid bars) and the single‐layer clocks (hatched bars). (g) Heatmap showing the effect of leaving one epigenetic layer out on the predictive accuracy of the pan‐epigenetic clock. Each circle denotes the percent change in age‐prediction performance (relative to the pan‐epigenetic clock trained on all epigenetic layers) assessed on the epigenetic mark on the *y*‐axis when the epigenetic mark on the *x*‐axis is left out from model training (Methods).

Having identified core genes at which aging reflects coordinated changes across epigenetic layers, we next asked whether these various layers are equally predictive of age. To address this question, we trained a collection of “single‐layer” clocks, each designed to estimate age using genes as input features, with values of each gene drawn from one particular epigenetic layer (Figure 3a). Of the seven layers, H3K36me3 and H3K9me3 were omitted from clock‐related analyses due to sparse coverage in mice, yielding a total of five epigenetic clock models (Figure S1c). Using a 10‐fold cross‐validation procedure to assess these models (Methods), we found that the prediction accuracy varied by epigenetic layer (lowest *ρ*: H3K4me1 = 0.54; highest *ρ*: DNAm = 0.91, Figure S4a–c). Training single‐layer clocks for increasing numbers of donors revealed that some marks had a greater age‐prediction capacity than others, even when the number of training donors was matched (Figure S4d). As the training sample size increased, H3K27me3 and DNAm showed the most rapid improvements in model performance (scaling rate: DNAm = 0.112, H3K27me3 = 0.109; Figure S4d; Methods), while H3K4me1 showed the slowest (H3K4me1 scaling rate = 0.056). On the other hand, each of these single‐layer clocks exhibited similar performance whether applied to humans or mice (Figure S4a–c), suggesting that, like DNA methylation (Lu et al. 2023; Wang et al. 2020), histone modifications follow an evolutionarily conserved trajectory of change during aging.

We next compared the results of the single‐layer clocks to a “pan‐epigenetic” clock designed to predict donor age from any of the five epigenetic layers analyzed (Figure 3a; Methods). This model had an architecture identical to the single‐layer clocks but was trained using profiles from all epigenetic layers. This pan‐epigenetic clock accurately predicted age in both humans (Spearman *ρ* = 0.67; Figure 3b) and mice (Spearman *ρ* = 0.80, Figure 3c) from any epigenetic mark (Figure 3d). Notably, the performance of the pan‐epigenetic clock closely matched that of the separate single‐layer clocks in each species (Spearman *ρ* = 0.64, *p* = 0.048; Figure 3e) and tissue (Spearman *ρ* = 0.95, *p* = 2.0 × 10^−6^; Figure 3f) when assessed on held‐out donors in 10‐fold cross‐validation. When we left out all profiles from one epigenetic layer during training, the model largely retained the ability to estimate the age of held‐out donors using that particular layer (Figure 3g; Methods). H3K4me1 had the smallest decrease in performance when left out (−23%), while H3K27me3 had the largest (−62%). These results demonstrated that the aging signals contained within each epigenetic layer are similar enough to be effectively represented by a single model.

### 2.4 Different Epigenetic Layers Exhibit Synchronized Aging Rates

**FIGURE 4:**
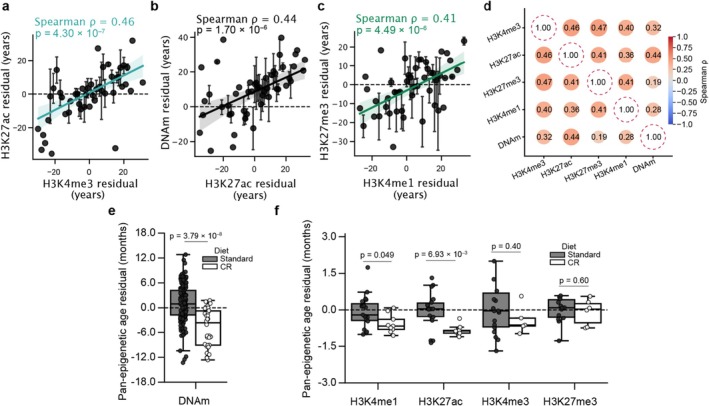
Synchronization of aging across epigenetic layers. (a) Scatter plot comparing age prediction residuals between H3K4me3 and H3K27ac (*n* = 109 human donors). Each point represents the average H3K27ac residual for all donors with the same (±1 year) H3K4me3 residual while error bars represent the standard error of this estimate. *ρ*, Spearman correlation between the two residuals. *p*‐value denotes significance calculated by modeling Spearman *ρ*'s as a Student's *t* distribution. (b) Similar to (a) but comparing H3K27ac and DNAm (c) Similar to (a) but comparing H3K4me1 and H3K27me3. (d) Correlation matrix illustrating the relationship between age prediction residuals (i.e., partial correlation; Methods) from the pan‐epigenetic clock across five epigenetic modifications (*n* = 109 human donors), with circle size and color representing the strength and direction of Spearman correlations. All pairwise correlations are significant (*p* < 0.05). *p*‐value denotes significance calculated by modeling Spearman *ρ*'s as a Student's *t* distribution. (e) Box plots depicting the pan‐epigenetic age residuals (in months) from the DNAm of mice under standard (*n* = 175 mice) versus caloric restriction (*n* = 32 mice) diets. Points indicate individual mice. *p* value calculated using a two‐sided Mann–Whitney *U* test. (f) Similar to (e), but age predictions made using histone marks (*n* = 69 control fed mice; *n* = 29 CR mice).

If there is a coordinated process driving epigenetic change across layers, individuals appearing older/younger than their chronological ages according to one layer would likewise appear older/younger according to other layers. To test this hypothesis, we applied the pan‐epigenetic clock to donors profiled for all five epigenetic modifications (*n* = 109 human donors). We found that the degree of over‐ or under‐prediction of chronological age was indeed synchronized across layers (Figure 4a–d; Methods). For instance, for individuals whose H3K27ac profile indicated that they were 1 year older than their chronological age, their H3K4me3 profile produced an overprediction of 0.52 ± 0.1 years (mean ± s.d., Figure 4a). This directional synchronization of the rate of epigenetic change between modifications was not limited to associations within histone marks, with DNAm patterns also yielding age predictions that were significantly associated with those of histone marks (Figure 4b,d).

Reduced methylation age is strongly promoted by chronic caloric restriction (CR) (Wang et al. 2017), which has long been known to extend lifespan in many species (Speakman and Mitchell 2011). We thus evaluated whether this intervention would likewise slow the progression of age‐related changes across the various layers of the epigenome. Applying the pan‐epigenetic clock to tissues from C57BL/6 mice fed a CR or control diet (Hillje et al. 2022; Petkovich et al. 2017), we found that every layer tended to produce younger age predictions in CR mice, but that these reductions reached statistical significance for only DNAm, H3K4me1, and H3K27ac (Figure 4e,f). Ages inferred from DNAm and H3K27ac profiles of CR mice were on average 4.6 and 0.9 months younger than their chronological ages, respectively. Thus, CR appears to exert a youth‐preserving influence on multiple layers of the epigenome.

## 3 Discussion

Collectively, our results support three general findings regarding the links between the multiple epigenetic marks and age. First, epigenetic layers in both humans and mice follow a shared trajectory of age‐related change—converging on the same genomic loci and advancing at synchronized rates (Figures 1 and 2). Second, this coherence allows a single pan‐epigenetic clock to predict chronological age from any layer in either species (Figure 3). Third, within each individual, the epigenetic age inferred from one mark is echoed by similar age predictions based on every other mark (Figure 4).

The genomic loci showing synchronized remodeling across all epigenetic layers (Figure 2) are strongly enriched for developmental genes that become epigenetically repressed with age—alongside inflammatory pathway genes that are activated. This pattern echoes findings from studies of a single epigenetic layer—DNA methylation—measured across 100 mammalian species (Lu et al. 2023) and across 17 human tissues (Jacques et al. 2025), which similarly report age‐related repression of developmental regulators. The coordinated epigenetic activation of inflammatory genes likewise reinforces previous results documenting a progressive increase in inflammatory signaling during aging, at the physiological (Ferrucci and Fabbri 2018) and molecular (Moqri, Poganik, et al. 2024) scales. Because aging affects the entire organism, we designed our study to integrate epigenetic profiles from numerous tissues; accordingly, the age‐related changes we report here are those generally conserved across tissues. Together, these observations suggest that the pan‐epigenetic change we observe across the mouse and human life course represents a cross‐tissue reorientation of the chromatin landscape away from developmental programs and toward inflammatory states during mammalian aging.

Recent evidence points to extensive interaction among epigenetic regulatory enzymes (Lempiäinen and Garcia 2023). Such crosstalk provides a potential molecular explanation underlying the coordination of age‐related changes we observed across epigenetic layers. For instance, DNA methyltransferase 1 (*DNMT1*) selectively methylates loci marked by H3K9me3 (Ren et al. 2020)—aligning with our finding of age‐associated DNAm gains at regions with increasing H3K9me3 (Figure 1d,g). Similarly, physical interaction between the histone acetyltransferase p300 and the SET1 family of methyltransferases promotes cooperative H3K4me3 and H3K27ac deposition (Tang et al. 2013), which is likewise reflected in our results (Figure 1f,i). Age‐related changes impacting one epigenetic layer may reverberate through the entire epigenetic network, advancing epigenetic age across all layers. Conversely, a therapeutic intervention that rejuvenates a single epigenetic layer could possibly restore youthful epigenetic states throughout multiple layers of the epigenome, similar to the effect we observed in calorically restricted mice (Figure 4e,f). Nevertheless, while all epigenetic layers measured in the same individual tended to agree in the over‐ or under‐prediction of their age, this association was not perfect (Spearman *ρ* = 0.19–0.47). Additionally, caloric restriction slowed age‐related epigenetic changes in DNAm, H3K4me1, and H3K27ac, but not H3K4me3 or H3K27me3. Thus there may be an extent to which different layers are affected by distinct processes and encode distinct aging information.

As to what are the initial causes of the observed pan‐epigenetic alterations, two main theories have emerged. The first, a developmental or “pseudo‐programmatic” model (Gems et al. 2024), posits that aging represents a continuation of the epigenetic trajectories established during development. This view is supported by the remarkable conservation of age‐associated epigenetic shifts across species and the enrichment of these changes for developmental genes (Lu et al. 2023; Moqri, Cipriano, et al. 2024; Wang et al. 2020). These patterns are supported by the present results, in which repressive epigenetic marks converge on genes governing development, in both mice and humans (Figure 2e,f). The second theory—the stochastic drift model—suggests that aging‐associated epigenetic alterations arise from the random accumulation of molecular errors and damage‐induced changes throughout the epigenome. Supporting this theory, computational simulations have demonstrated that much of the epigenetic change seen during aging can be explained by the random accumulation of epigenetic alterations (Meyer and Schumacher 2024; Tong et al. 2024), which may result from DNA damaging events (Koch et al. 2025; Yang, Hayano, et al. 2023). The two different theories are not strictly incompatible (Tarkhov et al. 2024), such that epigenetic aging may arise from both inherited developmental programs and the accumulation of molecular noise, with the epigenetic crosstalk reported here (Figure 1) amplifying alterations from either source.

Some limitations of this study are as follows: First, our results are based on cross‐sectional data. Future studies applying longitudinal profiling will be essential to disentangle true age‐related changes from cohort effects. Second, while this study has expanded the palette of epigenetic marks associated with aging, it is not exhaustive in this respect—inclusion of additional modifications and histone variants could refine the observed synchrony. Third, despite stringent batch correction (Methods), differences in the ChIP‐seq and WGBS protocols among the studies from which we drew data may affect our results. Fourth, some epigenetic layers had limited sample sizes in mice, particularly H3K9me3 and H3K36me3, limiting the statistical power to detect age‐related change in these layers.

## 4 Methods

### 4.1 Table of Analyzed Datasets

**Table:** 

Dataset name/description	Epigenetic layer	Species
Canadian Epigenetics, Environment and Health Research Consortium (CEEHRC) (Bujold et al. 2016)	DNA methylation, H3K4me3, H3K27ac, H3K27me3, H3K4me1, H3K36me3, H3K9me3	Human
ENCODE (ENCODE Project Consortium et al. 2020)	DNA methylation, H3K4me3, H3K27ac, H3K27me3, H3K4me1, H3K36me3, H3K9me3	Human
BLUEPRINT (Fernández et al. 2016)	DNA methylation, H3K4me3, H3K27ac, H3K27me3, H3K4me1, H3K36me3, H3K9me3	Human
Signal et al. (2024)	H3K4me3, H3K27ac, H3K27me3	Mouse
Yang, Occean, et al. (2023)	H3K27me3, H3K36me3, H3K9me3	Mouse
Stubbs et al. (2017)	DNA methylation	Mouse
Hillje et al. (2022)	H3K4me3, H3K27ac, H3K27me3, H3K4me1	Mouse
Meer et al. (2018)	DNA methylation	Mouse
Petkovich et al. (2017)	DNA methylation	Mouse

### 4.2 Histone Modification Processing

ChIP–seq narrowPeak files were gathered from six public datasets—ENCODE, BLUEPRINT, CEEHRC, Signal et al. (2024), Yang, Occean, et al. (2023), and Hillje et al. (2022). The locations of autosomal gene bodies in humans and mice were retrieved from the Ensembl v109 catalog (Harrison et al. 2024). Then, for each histone profile separately, the mean narrowPeak score of all peaks overlapping a given gene was taken as the epigenetic value of that gene, while genes without any overlapping peaks in that sample were assigned a value of zero. Mouse gene identifiers were converted to their one‐to‐one human orthologs via *pybiomart* (Smedley et al. 2009), placing every profile in a common human‐gene coordinate system. Genes with no overlapping peaks in any sample were discarded. This procedure yielded gene‐level histone modification profiles aligned between species.

### 4.3 DNA Methylation Processing

Whole‐genome bisulfite sequencing files were obtained from six public datasets—ENCODE, BLUEPRINT, CEEHRC, Stubbs et al. (2017), Meer et al. (2018), and Petkovich et al. (2017). CpG sites were intersected with autosomal gene bodies defined in Ensembl v109 via *pybedtools* (Dale et al. 2011). For each sample, the mean methylation fraction of all CpGs falling within a given gene was recorded. Genes lacking overlapping CpGs were assigned a value of zero. Mouse gene identifiers were mapped to their one‐to‐one human orthologs using *pybiomart* (Smedley et al. 2009), placing every methylome on the same human‐gene coordinate system.

### 4.4 Integration and Normalization of Epigenetic Layers

Gene‐level histone mark and DNA methylation datasets were first scaled separately. Within each {dataset × epigenetic‐mark} stratum: epigenetic signal values were min–max normalized to the interval [0, 1] and, for DNA methylation datasets, missing values (< 0.5% of all values) were imputed as the mean across all other samples. To mitigate inter‐study batch effects, each dataset was inverse normal transformed (INT) (McCaw et al. 2020). The epigenetic signal values of each gene were ranked across samples; these ranks were converted to uniform percentiles, and the inverse standard normal cumulative distribution function was applied to map these percentiles to *z*‐scores. During the training of all machine learning models, INT and mean‐imputation were instead performed within each cross‐validation fold, to prevent information leakage across folds. In each cross‐validation fold, the INT and mean‐imputation parameters from the training set were applied to the held‐out test data before prediction. After normalization, all datasets, including both histone marks and DNA methylation, were combined and genes for which epigenetic values were present in all samples were retained (*n* = 17,602).

### 4.5 Overlap of Age‐Associated Regions Across Epigenetic Layers and Species

Within the batch‐corrected matrix, gene‐wise associations between chronological age and epigenetic values were quantified separately for every {species × epigenetic‐mark} stratum using Spearman correlation. Ranking the correlation values by their unsigned magnitude, pairwise overlaps among the 1000 genes with the strongest age association were computed for all histone‐mark combinations within each species (Figure 1d,e). The expected overlap under random expectation was taken to be:

$$\text{Expected overlap}=N_{\mathrm{top}}\times {\left[\frac{N_{\mathrm{top}}}{G}\right]}^{2}$$

where $N_{\mathrm{top}}$ = 1000 genes and G = 17,602 (the total number of genes). Fold enrichment was calculated as the observed divided by expected overlap. Statistical significance was assessed with two‐sided binomial tests followed by Bonferroni correction. Concordance between human and mouse age‐associated genes was evaluated analogously (Figure 1f).

### 4.6 Interaction Between Age‐Related Changes Among Epigenetic Layers

For each pair of epigenetic layers *A* and *B*: (1) the 1000 genes with the strongest positive (age‐increasing set) or negative (age‐decreasing set) age‐association (Spearman *ρ*) in layer *A* were selected; (2) the distribution of Spearman *ρ*'s in layer *B* was extracted for the genes in the age‐increasing and age‐decreasing sets of layer *A*; (3) the median difference in Spearman *ρ*'s between these sets (age‐increasing—age‐decreasing) was calculated; and (4) a two‐sided Mann–Whitney *U* test was used to assess if there existed statistical significance of medians between the distribution of Spearman *ρ* values in the age‐increasing versus age‐decreasing sets. These *p* values were Bonferroni‐corrected across all (7 choose 2=) 21 comparisons (*α* = 0.05). A significant median difference signified cross‐talk between epigenetic layers, with positive values indicating that genes gaining signal in layer A with age also tend to gain signal in layer B, whereas negative values denoted opposing trends (Figures 1g–i and S3a–d).

### 4.7 Enrichment Analyses

Genes showing the strongest age‐associated epigenetic repression were defined by taking the intersection of the top 1000 most positively age‐associated genes according to each repressive mark (H3K27me3, H3K9me3, DNAm) and the top 1000 most negatively age‐associated genes according to each activating mark (H3K27ac, H3K36me3, H3K4me1, H3K4me3) in each species (*n* = 778 genes). The converse was done to define the most epigenetically activated genes with age (*n* = 427 genes). Enrichments of these genes for Gene Ontology Biological Process and Reactome pathways were measured using the Enrichr API via the gseapy (Fang et al. 2023) package, testing against the “GO_Biological_Process_2023” and “Reactome_Pathways_2024” libraries, respectively. The universe for each test comprised all genes quantified in the age–correlation analysis. For each gene set (repressed or activated), terms containing fewer than two genes or with Benjamini–Hochberg–adjusted *p* ≥ 0.05 were excluded. The Combined Score from Enrichr (log *p* × *z*‐score) was used as the enrichment metric.

### 4.8 Representation of Chronological Age

Developmental and aging time points were aligned between humans and mice by representing the age of each donor as a percentage of the maximum lifespan of its respective species, as described by Lu et al. (2023). Briefly, each donor's age (in years) was divided by the species‐specific maximum lifespan reported in GenAge (de Magalhães et al. 2024) (120 years for humans; 4 years for mice) to obtain a unitless relative age between 0 and 1. These values were then transformed with the negative log–log transformation:

$$\text{Scaled}\;\mathrm{age}=-\log\left[-\log\left(\frac{\text{chronological}\;\mathrm{age}}{\text{maximum lifespan}}\right)\right]$$

The reverse transformation was used to present values in units of years in all figures.

### 4.9 Clock Dataset

Epigenetic profiles originating from cell lines, sex‐specific tissues (prostate, breast or other reproductive organs), placenta and any tissue represented by < 50 libraries were excluded from both model training and evaluation. Calorically restricted samples (*n* = 298 mice) were likewise withheld from model fitting but retained for subsequent inference. Due to their limited representation in mice (Figure S1c), H3K36me3 and H3K9me3 profiles were omitted from all clock construction steps, leaving five epigenetic layers (H3K4me3, H3K27ac, H3K27me3, H3K4me1, and DNA‐methylation). The resulting dataset comprised 2598 profiles from 1005 unique donors.

### 4.10 Single‐Layer Clock Training

Five independent gradient boosted decision‐tree regressors (Chen and Guestrin 2016) were fit—one for each epigenetic layer (H3K4me3, H3K27ac, H3K27me3, H3K4me1 and DNA‐methylation) to predict the age of mouse and human samples. Models used the 17,602 genes retained after layer integration as predictors (see “Integration and Normalization of Epigenetic Layers”); no additional covariates were included. Training employed stratified ten‐fold cross‐validation in which the number of profiles from donors of each age quintile was balanced across folds, while ensuring that all profiles from the same donor resided in a single fold. Default XGBoost hyper‐parameters were used, and performance was computed over the ten held‐out folds. Within each training fold, we retained only features whose association with age, quantified by Spearman's *ρ* in human and mouse samples separately, was stronger than |*ρ*| > 0.2 in both species and had the same sign in humans and mice.

### 4.11 Pan‐Epigenetic Clock Training

The pan‐epigenetic clock was likewise an XGBoost model, trained in an identical way to the single‐layer clocks. The same feature set, feature selection, and samples were used. However, instead of samples from each epigenetic layer being trained on and predicted by separate clocks, a single model was trained over all profiles in 10‐fold CV.

### 4.12 Leave‐One‐Layer‐Out Analysis of Cross‐Layer Learning

To determine whether the pan‐epigenetic clock leverages shared aging information across layers, we retrained the model five times, each time leaving all profiles of one epigenetic layer out of the training set and performing 5‐fold CV on the remaining samples. Then, in each CV fold, the performance of the model was evaluated on (1) all samples from the left‐out layer and (2) the held‐out samples from the layers used in training.

### 4.13 Scaling Rate of Age‐Prediction

Using donors from the CEEHRC, ENCODE, and BLUEPRINT datasets who had been profiled for multiple epigenetic marks (*n* = 219 donors), single‐layer clocks were trained on training cohorts of varying size. For each mark, we repeatedly (ten times) sampled 20–200 donors in 20‐donor increments without replacement, trained a single‐layer clock on each training set exactly as described above, and recorded the age‐prediction accuracy (Spearman *ρ*). The scaling rate for a given epigenetic layer was taken as the slope of a zero‐intercept ordinary‐least‐squares regression of these Spearman *ρ* values on the log_10_‐transformed number of training donors. The log transformation was used as the scaling rate was nonlinear.

### 4.14 Software

All analyses were performed in Python 3.10. Data analysis was conducted using Pandas 1.5.3, SciPy 1.10.0, Pingouin 0.5.3, and Statsmodels 0.13.5. Data were visualized with Seaborn 0.12.1 and Matplotlib 3.7.1.

## Author Contributions

Z.K. and A.L. designed the study, carried out the primary data analyses, and wrote the manuscript. T.I. designed the study and wrote the manuscript.

## Funding

This study was funded by the National Institutes of Health under awards U54 CA274502 and P41 GM103504.

## Conflicts of Interest

T.I. is a co‐founder, member of the advisory board, and has an equity interest in Data4Cure and Serinus Biosciences. T.I. is a consultant for and has an equity interest in Ideaya Biosciences and Eikon Therapeutics. The terms of these arrangements have been reviewed and approved by the University of California San Diego in accordance with its conflict‐of‐interest policies.

## Supporting information


**Figure S1:** acel70380‐sup‐0001‐Figures.docx.

## Data Availability

Data can be accessed through the respective publications: Canadian Epigenetics, Environment and Health Research Consortium (CEEHRC) (Bujold et al. 2016), ENCODE (ENCODE Project Consortium et al. 2020), Blueprint (Fernández et al. 2016), Signal et al. (2024), Yang, Occean, et al. (2023), Stubbs et al. (2017), Hillje et al. (2022), Meer et al. (2018), Petkovich et al. (2017). All custom algorithms and analysis code is in the GitHub repository at https://github.com/zanekoch/pan‐epigenetic‐age‐prediction.
