# Supplementary material for: Pan‐Epigenetic Age Prediction in Mammals
Source: Aging Cell. 2026 Jan 27;25(2):e70380. doi: 10.1111/acel.70380 (PMC12841597; doi:10.1111/acel.70380)
Supplement: Supplementary file 1 — Figure S1: acel70380‐sup‐0001‐Figures.docx. [file ACEL-25-e70380-s001.docx]

## **Supplementary Figures**

###
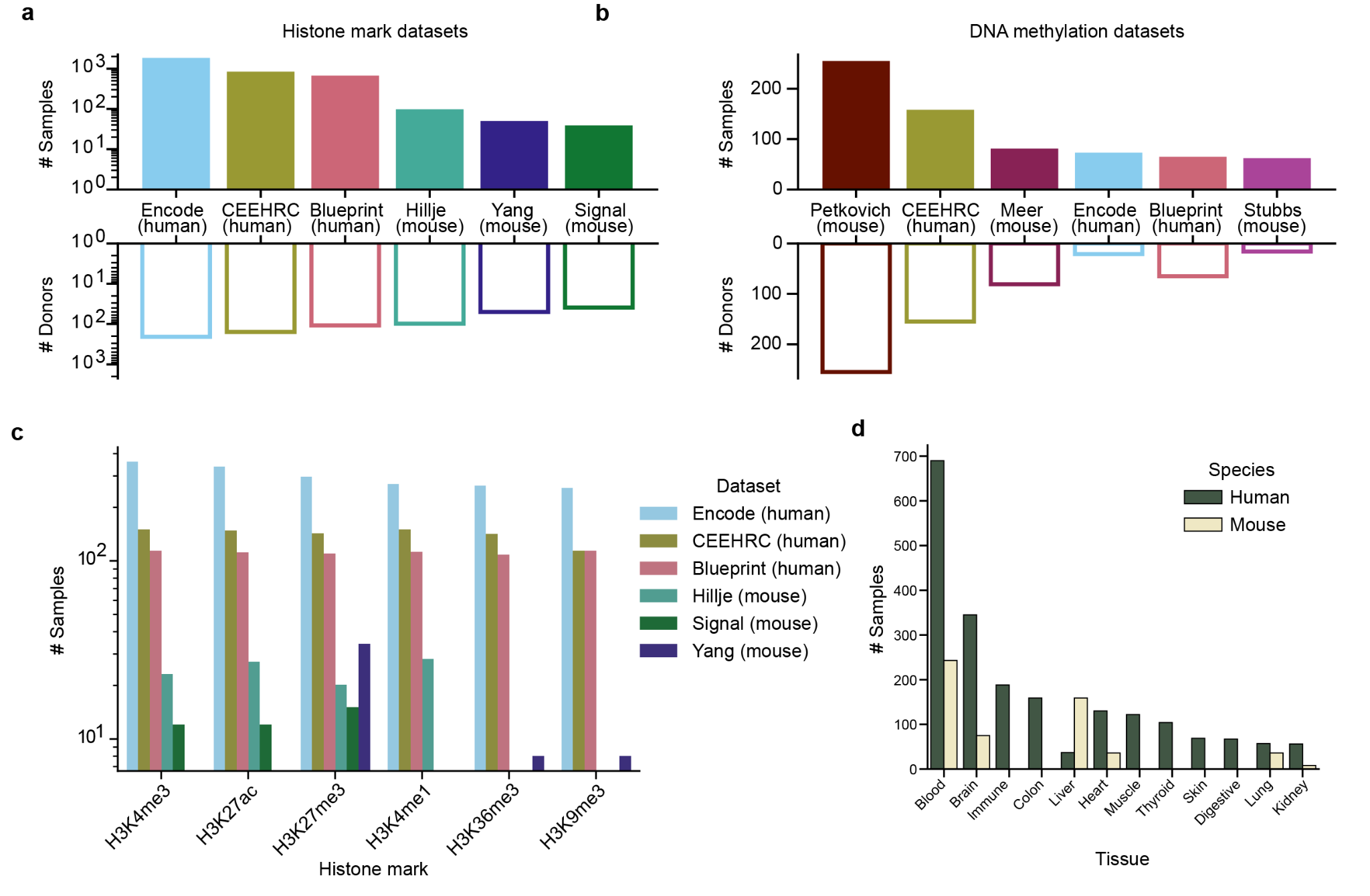
[Supplementary Figure 1](#suppl_data_characteristics): Dataset overview

**a)** Bar plots summarizing the number of profiles and unique donors (bottom) for each histone‐mark dataset in human (Encode, CEEHRC, Blueprint) and mouse (Hillje, Yang, Signal). Y‐axes are on a log₁₀ scale.

**b)** Bar plots summarizing the number of DNA methylation profiles (top) and unique donors (bottom) for each dataset in human (CEEHRC, Encode, Blueprint, Me­er, Stubbs) and mouse (Petkovich). Donor counts are shown on a log₁₀ scale.

**c)** For each histone modification (x‐axis), the total number of samples contributed by each dataset, with profiles per mark plotted on a log₁₀ y‐axis.

**d)** Bar plot of the number of samples per tissue, stratified by species.

###
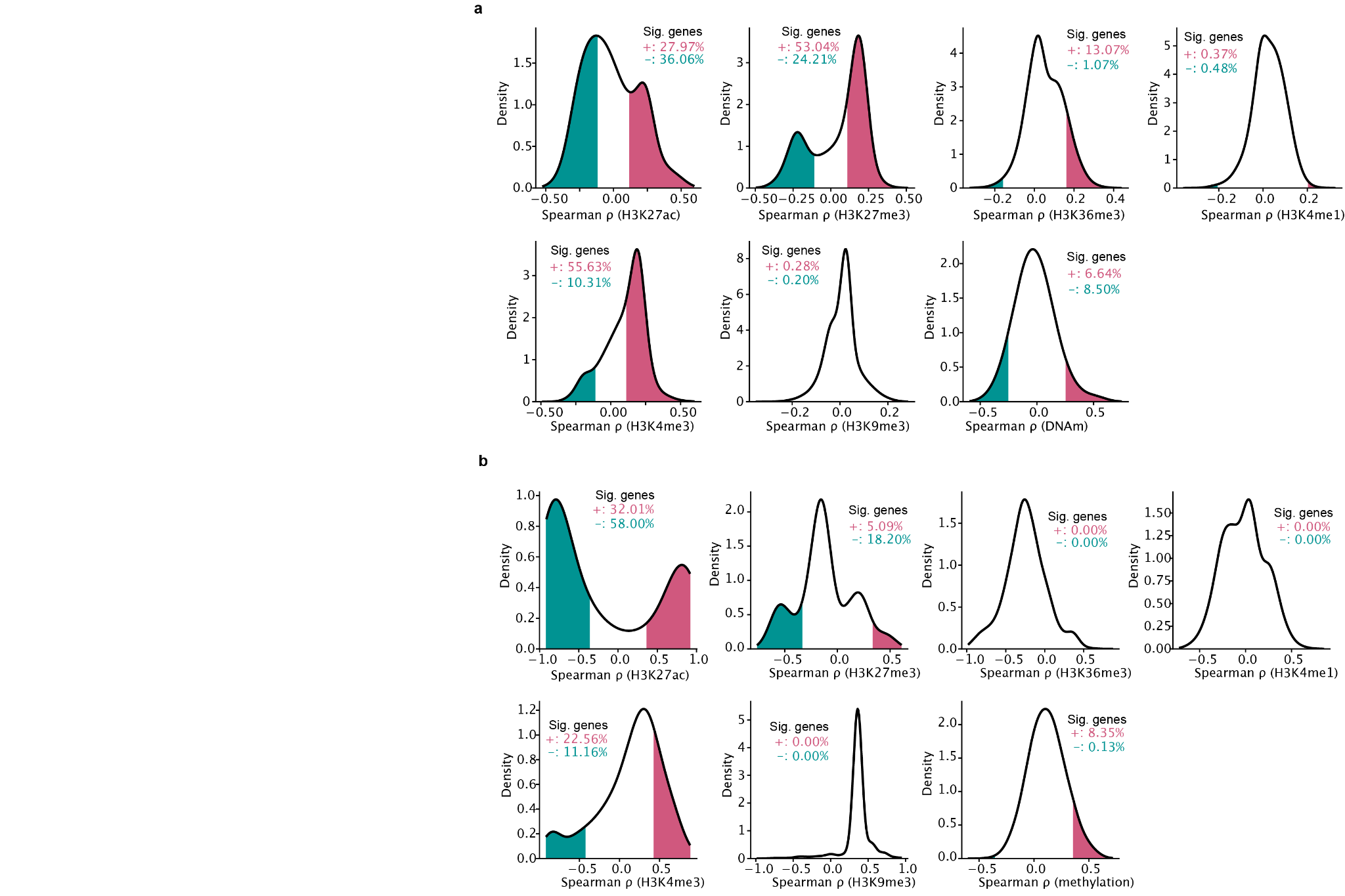


### [Supplementary Figure 2](#suppl_age_change): Age-related changes in each epigenetic layer

**a)** Kernel density estimates of the distribution of Spearman’s ρ between gene-level epigenetic signal (n = 17,602 genes) and age across all human donors (n = 482 donors). Regions shaded in magenta and teal indicate genes with significantly positive or negative age associations (FDR < 0.05), respectively. The text on the figure denotes the percentage of genes with significantly increasing (+) or decreasing (-) epigenetic signal.

**b)** Equivalent kernel density estimates for mouse donors (n = 523 donors), plotted as in (a).


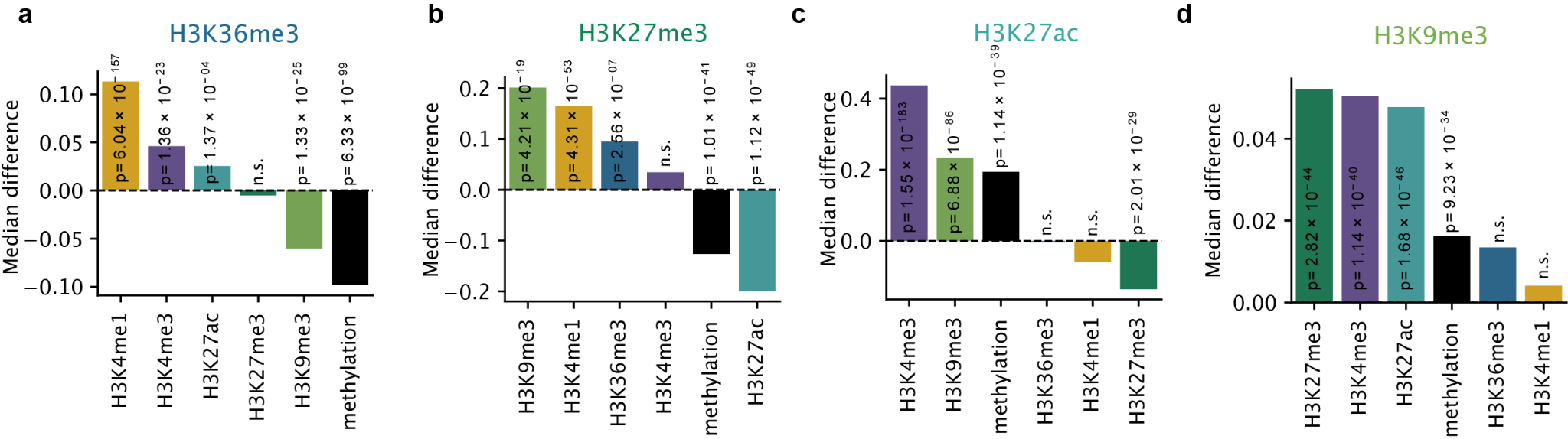


### [Supplementary Figure 3](#suppl_interaction): Interaction of age-related changes among epigenetic layers

**a-d)**, For each focal mark: H3K36me3 (**a**), H3K27me3 (**b**), H3K27ac (**c**) and H3K9me3 (**d**), genes were ranked by their Spearman age-association (ρ) according to each epigenetic layer shown on the x-axis and split into the 1,000 most positively and 1,000 most negatively associated. Bars show the median difference in ρ of the focal mark between these two gene sets. Positive values indicate that genes gaining signal in the x-axis layer with age also gain the focal mark, whereas negative values indicate the opposite. P values (two-sided Mann–Whitney U test) are shown above each bar; “n.s.” denotes p ≥ 0.01.

###
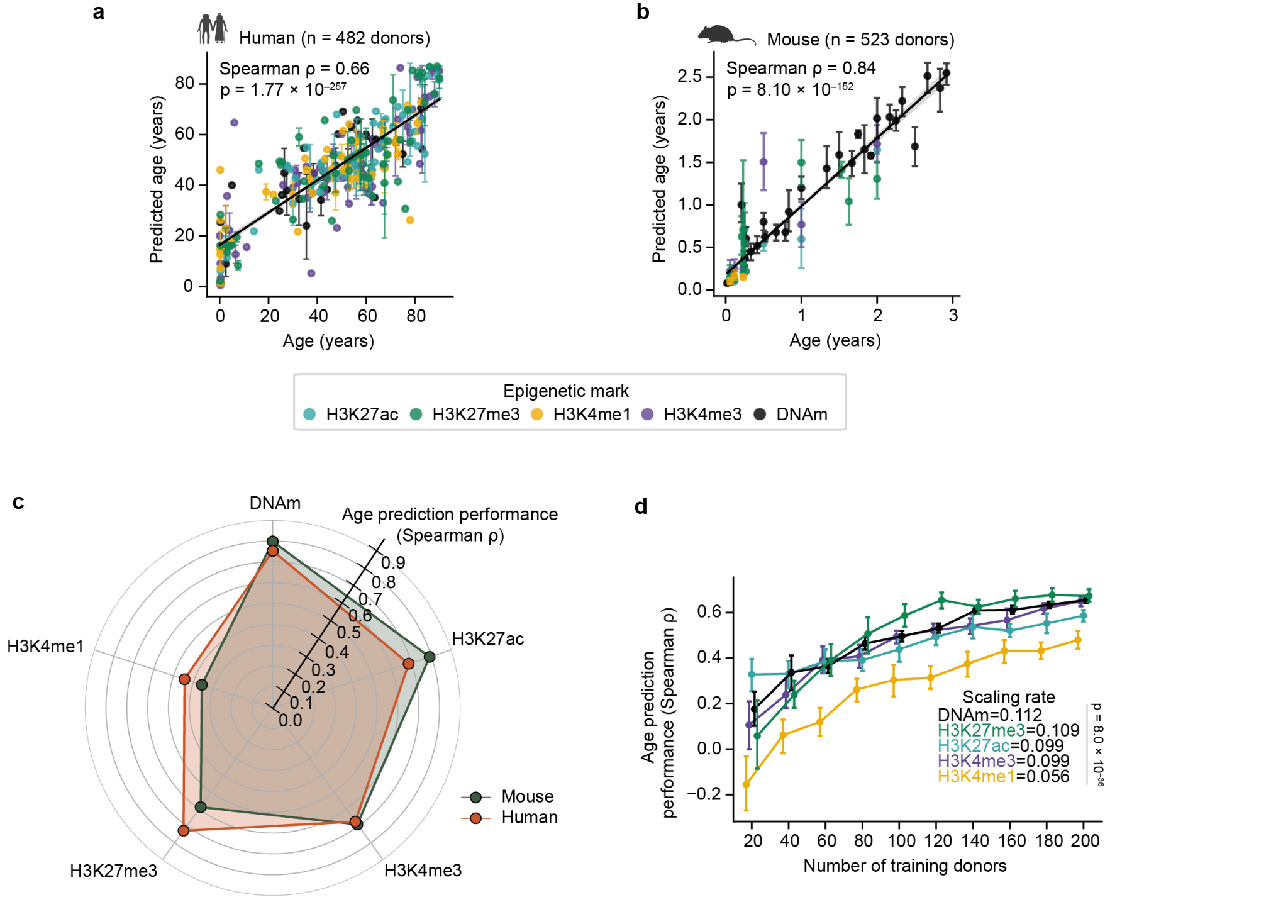


### [Supplementary Figure 4](#suppl_singlemark): Single-layer clock performance

**a)** A scatter plot of predicted versus actual age for human donors (n = 482 donors, n = 2,029 profiles) using the pan‐epigenetic clock. Each point represents an individual donor and errors bars the standard deviation of age predictions across samples from each tissue profiled in that donor. Predictions based on different epigenetic marks are shown in different colors.

**b)** Similar to (b) but for mouse donors (n = 523 mice, n = 569 profiles).

**c)** A radar plot comparing the predictive performance (Spearman ρ) of each single-layer clock on samples of each species (human: orange polygon, n = 482 donors; mouse: grey polygon, n = 523 donors).

**d)** A line plot showing age‐prediction performance of single-mark clocks as a function of the number donors in the training set. Points indicate the mean and standard deviation of the Spearman correlation between predicted and actual age for each clock trained on each number of donors, across 10 bootstrapped iterations of clock training. Scaling rate is defined as the change in age prediction performance, as measured by Spearman correlation, per log_10_ increase in the number of donors used in model training (**Methods**). P value shown for a Krustkal-Wallis test testing for a difference of age-prediction performance between layers.
